# Expression of MAGE-A and NY-ESO-1 cancer/testis antigens in medullary breast cancer: a retrospective immunohistochemical study

**DOI:** 10.3325/cmj.2011.52.171

**Published:** 2011-04

**Authors:** Božica Matković, Antonio Juretić, Giulio C Spagnoli, Viktor Šeparović, Marija Gamulin, Robert Šeparović, Nera Šarić, Martina Bašić-Koretić, Irena Novosel, Božo Krušlin

**Affiliations:** 1 University Hospital for Tumors, Zagreb, Croatia; 2 University of Zagreb, School of Medicine Zagreb, and Zagreb University Hospital Center, Zagreb, Croatia; 3 Institute of Research and Hospital Management, University of Basel, Basel, Switzerland; 4 Zagreb University Hospital Center, Zagreb, Croatia; 5 Dr. I. Pedišić General Hospital, Sisak, Croatia; 6 University of Zagreb, School of Medicine Zagreb and Sisters of Mercy University Hospital, Zagreb, Croatia; * The first two authors contributed equally to this study.

## Abstract

**Aim:**

To immunohistochemically evaluate the expression of MAGE-A1, MAGE-A, and NY-ESO-1 cancer/testis (C/T) tumor antigens in medullary breast cancer (MBC) tumor samples and to analyze it in relation to the clinicopathological features.

**Methods:**

This retrospective study included samples from 49 patients: 40 with typical MBC and 9 with atypical MBC. Tumor specimens were obtained from patients operated on in the University Hospital for Tumors and the Sisters of Mercy University Hospital, Zagreb, Croatia, from 1999 to 2005. Standard immunohistochemistry was used on archival paraffin-embedded MBC tissues.

**Results:**

MAGE-A1, MAGE-A, and NY-ESO-1 antigens were expressed in 33% (16/49), 33% (16/49), and 22% (11/49) of patients, respectively. No difference between the groups with and without C/T tumor antigen expression in age at diagnosis, tumor size, axillary lymph node metastasis, adjuvant therapy, and HER-2 expression was identified. Significantly more patients died in the MAGE-A-positive group than in the MAGE-A-negative group (*P* = 0.010), whereas a borderline significance was found between MAGE-A1-positive and the MAGE-A1-negative group (*P* = 0.079) and between NY-ESO-1-positive and NY-ESO-1-negative group (*P* = 0.117). Overall survival, as evaluated by the Kaplan-Meier curves, was lower in MAGE-A1- (*P* = 0.031), MAGE-A- (*P* = 0.004), NY-ESO-1-positive groups (*P* = 0.077).

**Conclusion:**

Expression of C/T antigens may represent a marker of potential prognostic relevance in MBC.

Breast cancers are a very heterogeneous group of diseases in terms of natural history, histopathological features, genetic alterations, gene-expression profiles, and response to treatment (1-5). Medullary breast cancers (MBC), both typical and atypical, account for <2% of breast invasive carcinomas. Despite histopathologically highly malignant characteristics, operable and non-metastatic MBCs have a more favorable prognosis than the more common infiltrating ductal breast carcinoma of the same stage (1,6-13). Recent updating of breast cancer classification, based on gene expression profile analyses, has indicated that MBCs can be considered as part of the basal-like carcinoma spectrum made up of the estrogen receptor (ER) negative-, progesterone receptor (PR) negative-, and human epidermal growth factor receptor 2 (HER-2)-negative tumors (‘triple-negative phenotype’) (14-17).

Cancer/testis (C/T) antigens are a subgroup of tumor-associated antigens expressed in normal testis germ line cells and trophoblast, and in various malignancies of different histological types. They were discovered in the last two decades by a combination of immunological and molecular biology techniques. Most genes that encode these antigens are localized on the X-chromosome, frequently as multigene families and are referred to as CT-X genes or CT-X antigens (18-23). Biological functions of C/T genes and C/T antigens in both germ lines and tumors remain poorly understood. Due to their tumor-associated expression pattern and limited presence in normal tissues, C/T antigens appear to be valuable targets for immunotherapy of cancer. The best-studied C/T antigens are those of the MAGE-A family and the NY-ESO-1 antigen (18-23). Our initial reports on C/T antigens expression detected by immunohistochemistry in breast invasive ductal carcinomas of no special type (24,25) has been confirmed by other studies (26,27). However, these studies have not been performed on special or relatively rare histological types of breast cancers, such as the MBC.

We have recently reported clinicopathological features of MBCs in 48 patients who were operated on in our two hospitals between 1999 and 2005 (28). The present study includes immunohistochemical analysis of the expression of C/T antigens MAGE-A, MAGE-A1, and NY-ESO 1 in these MBC samples.

## Patients and methods

This retrospective study included samples from 49 patients: 40 with typical and 9 with atypical MBC (28). Tumor specimens were obtained from patients operated on in the University Hospital for Tumors and the Sisters of Mercy University Hospital, Zagreb, Croatia, from 1999 to 2005. The patients were identified retrospectively in 2006 from pathological reports from the Departments of Pathology of the two hospitals. At the time of diagnosis, patients had nonmetastatic MBC (M0). Adjuvant therapy data were obtained from patients’ medical reports from the hospitals’ Oncology Departments (Table 1). The patients’ survival data were obtained at the end of 2008 from the Croatian National Cancer Registry and through personal contacts with patients and their physicians. The data on the disease-free survival were not available. The study protocol was approved by the Ethics Committee of the hospitals (28).

**Table 1:** Patients’ and medullary breast cancer characteristics

Characteristics, No. (%)	Medullary breast cancer patients (n = 49)
Age at diagnosis (years; median, range)	51 (28-82)
Type of surgery:	
mastectomy with axillary dissection	29 (59)
segmentectomy with axillary dissection	20 (41)
Tumor size (cm; median, range)	2.4 (0.8-5.0)
pT1	20 (41)
pT2	28 (57)
pT3	1 (2)
pT4	0
Axillary lymph node metastasis	
no	32 (65)
yes	17 (35)
Adjuvant therapy*:	
no	2 (4)
radiotherapy	12 (24)
chemotherapy	23 (47)
chemotherapy + radiotherapy	12 (24)
Survival (follow-up time in months; median, range):	68 (8-164)
alive	43 (88)
dead	6 (12)
dead (follow-up time in months; median, range)	42.5 (13-53)
Estrogen receptor:	
negative	46 (94)
positive	3 (6)
Progesterone receptor:	
negative	41 (84)
positive	8 (16)
Human epidermal growth factor receptor 2:	
negative (-, + or ++)	35 (71)
positive (+++)	14 (29)
MAGE-A1:	
negative (-)	33 (67)
positive (+,++,+++)	16 (33)
MAGE-A:	
negative (-)	33 (67)
positive (+,++,+++)	16 (33)
NY-ESO 1:	
negative (-)	38 (78)
positive (+,++,+++)	11 (22)

*At that time, adjuvant trastuzumab was not a standard therapy for the HER-2 +++ positive patients, ie, a therapy covered by our national health insurance system.

**Figure 1:**
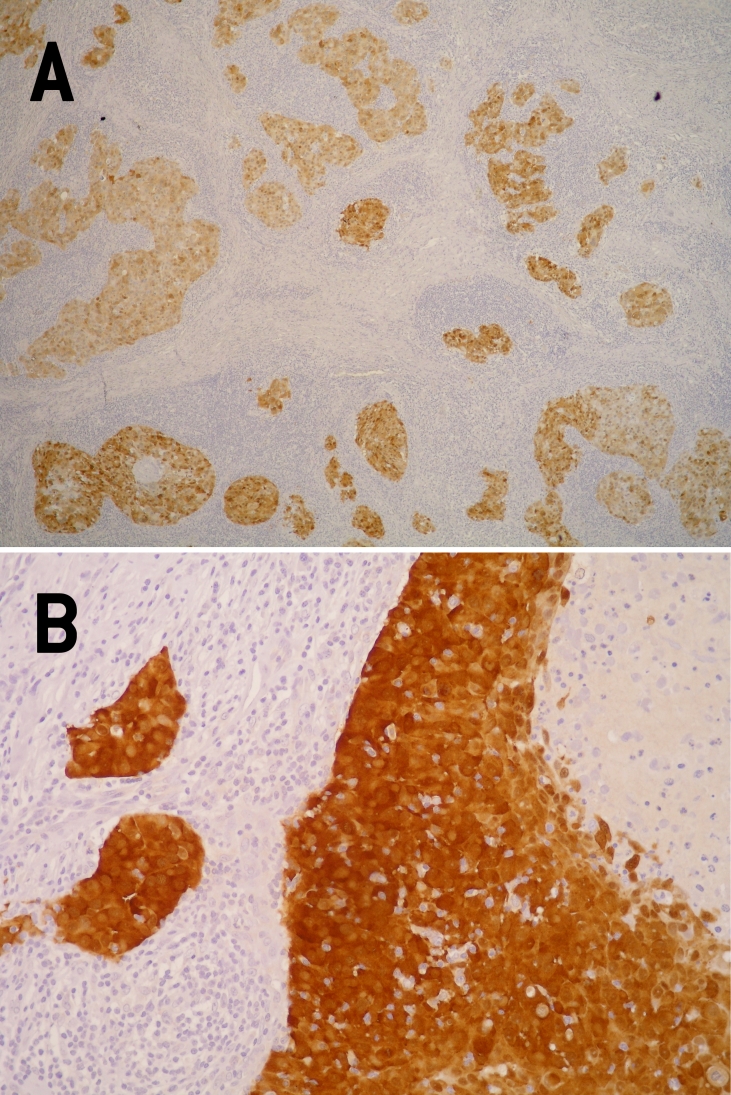
Immunohistochemical staining of MAGE-A1 and MAGE-A in medullary breast cancer tissues. (**A**) MAGE-A1 positive staining by monoclonal antibody 77B (PAP 40 × ). (**B**) MAGE-A positive staining by monoclonal antibody 57B (PAP 200 × ).

For routine histological analysis, the resected breast tissue was fixed immediately after surgery in 10% buffered formalin and later embedded in paraffin. From paraffin-embedded tumor samples, 4-μm thick sections were cut and stained with hematoxylin and eosin, and reviewed by the experienced pathologist (VS, BK, IN) in order to establish the diagnosis. Immunohistochemical staining was performed by appropriate monoclonal antibodies (mab) in accordance with the manufacturer’s instructions, as previously reported (19,24,25,28). Mab 1D5 (M7047, Dako, Glostrup, Denmark) and 1A6 (M3569 Dako) were used to detect ER and PR receptors, respectively. The DAKO Hercept TestTM kit (polyclonal antibody DA485, K5206, Dako), approved by the Food and Drug Administration, was used to detect HER-2. Immunohistochemical staining was performed following the Microwave Streptavidin Immuno Peroxidase protocol on DAKO TechMate Horizon automated immunostainer. Immunohistochemistry for C/T antigens MAGE-A1 (mab 77B) (29), multi MAGE-A (mab 57B) (30), and NY-ESO-1 (mab B9.8.1) (31) was performed following the same procedure. Positive staining for ER and PR was defined as nuclear staining in ≥10% of tumor cells, while positive staining for HER-2 was defined based on the percentage of tumor cells and the intensity of membrane staining. HER-2 immunostaning was considered positive when strong (+++) membranous staining was observed in at least 10% of tumor cells, whereas cases with 0 to ++ were regarded as negative (19,28). The staining for MAGE-A1, MAGE-A, and NY-ESO-1 was defined as weak with positive reaction when there were ≤10% of tumor cells (+), moderate with positive reaction when there were between 10 and 50% of tumor cells (++), and strong with positive reaction (+++) when there were >50% of tumor cells (Table 1) (24,25,28). Representative examples of immunohistochemical staining obtained with these mabs are presented in Figure 1.

Statistical analysis was performed using Statistics 5.5 software package (StatSoft, Inc., Tulsa, OK, USA). χ^2^ test was used for the group difference analysis of qualitative features, Fischer exact test for variables with low frequencies, nonparametric Mann-Whitney U test for numeric variables, and Kaplan-Meier curves with log-rank test for survival analysis. *P* values <0.05 were considered significant.

## Results and discussion

Patients’ median age was 51 years (range, 28-82 years). Modified radical mastectomy was the predominant operational procedure (29/49; 59%). The median tumor size was 2.4 cm (range, 0.8-5.0 cm) and axillary lymph node metastases were found in 17 out of 49 patients (35%). pT1 tumor size was found in 20, pT2 in 28, and pT3 in 1 patient. The majority of patients were ER- and PR-negative (94% and 84%, respectively). Twenty nine percent of patients (14 out of 49) were HER-2 antigen strongly positive (+++). Adjuvant therapy was applied in all but 2 patients (4%). The mean follow-up time of patients was 68 months (range, 8 to 164 months) and 43 out of 49 patients survived (88%). MAGE-A1, multi MAGE-A, and NY-ESO-1 specific staining was detectable in 33% (16/49), 33% (16/49), and 22% (11/49) of patients, respectively (Table 1).

**Figure 2:**
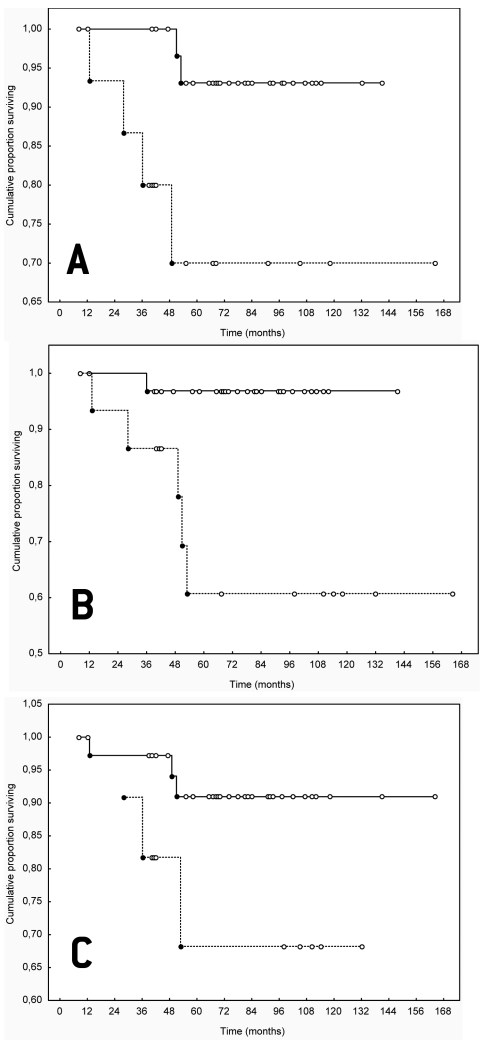
Kaplan-Meier survival curves of the expression of MAGE-A1, log rank test; *P* = 0.031 (**A**), MAGE-A, log rank test; *P* = 0.004 (**B**), and NY-ESO-1, log rank test; *P* = 0.077 (**C**) in medullary breast cancer tissues. Closed circle – dead patients; open circle – alive patients; full line – negative expression; interrupted line – positive expression.

Groups with and without expression of MAGE-A1, multi MAGE-A, and NY-ESO-1 were compared according to the following MBC clinicopathological features: patient’s age at diagnosis, type of operation, tumor size, presence of axillary lymph node metastasis, adjuvant therapy (chemotherapy and radiotherapy), HER-2 expression, and patient's survival. There was no difference between groups with and without C/T tumor antigen expression in these clinicopathologic parameters. Compared with MAGE-A-negative group, significantly greater number of patients died in the MAGE-A-positive group (*P* = 0.010), whereas borderline significance was found between the MAGE-A1-positive and MAGE-A1- negative group (*P* = 0.079) and betweenNY-ESO-1-positive and NY-ESO-1-negative group (*P* = 0.117). Overall survival of patients with MBC, as evaluated by the Kaplan-Meier curves, was lower in the groups expressing C/T antigens (Table 2). In particular, MAGE-A1-positive group had a significantly lower overall survival (*P* = 0.031, log-rank test) than MAGE-A1-negative group (Figure 2A). Similarly, multi MAGE-A positivity was also associated with a significantly lower overall survival (*P* = 0.004) (Figure 2B). A similar trend was also detectable for NY-ESO-1 (*P* = 0.077), although the difference did not reach significance (Figure 2C).

**Table 2:** Comparison of groups with and without MAGE-A1, MAGE-A, and NY-ESO-1 expression according to medullary breast cancer clinicopathological features

	No. (%) of patients with tumor expressing								
	MAGE-A1			MAGE-A			NY-ESO-1		
Characteristics	negative, n = 33 (67)	positive, n = 16 (33)	*P*	negative, n = 33 (67)	positive, n = 16 (33)	*P*	negative, n = 38 (78)	positive, n = 11 (22)	*P*
Age (years; median, range)	52 (28-69)	48.5 (32-82)	0.847^‡^	53 (28-69)	47.5 (32-82)	0.423^‡^	52 (34-82)	49 (28-61)	0.110^‡^
Tumor size (cm; median, range)	2.4 (0.8-5.0)	2.3 (0.8-4.0)	0.659^‡^	2.3 (0.8-5.0)	2.5 (1.2-4.0)	0.165^‡^	2.3 (0.8-5.0)	2.5 (1.1-4.0)	0.499^‡^
Axillary lymph node metastasis	12 (36)	5 (31)	0.973*	13 (39)	4 (25)	0.501*	15 (40)	2 (18)	0.287^†^
Adjuvant chemotherapy	22 (67)	13 (81)	0.336^†^	25 (76)	10 (63)	0.501^†^	28 (74)	7 (64)	0.705^†^
Adjuvant radiotherapy	15 (46)	9 (56)	0.686*	18 (55)	6 (38)	0.415*	19 (50)	5 (46)	0.938*
Estrogen receptor positive	2 (6)	1 (6)	-	3 (9)	0 (0)	-	3 (8)	0 (0)	-
Progesterone receptor positive	7 (21)	1 (6)	0.245^†^	7 (21)	1 (6)	0.245^†^	7 (18)	1 (9)	0.663^†^
Human epidermal growth factor receptor 2 positive (+++)	9 (27)	5 (31)	1.000^†^	7 (21)	7 (44)	0.175^†^	11 (29)	3 (27)	1.000^†^
No. of dead patients	2 (6)	4 (25)	0.079^†^	1 (3)	5 (31)	*0.010^†^*	3 (8)	3 (27)	0.117^†^

*χ^2^ test.

†Fischer exact test.

‡Mann-Whitney U test; significant values are italicized.

Our study suggested that the studied C/T antigens may be used in MBC as tumor markers of potential prognostic relevance. Due to the relative rarity of this type of breast cancer, in order to obtain a final confirmation of this observation, the expression of these C/T antigens needs to be investigated on a greater number of tumor samples. Interestingly, however, a recent publications by Grigoriadis et al (27) and Curigliano et al (32) have pointed out that the expression of CT-X antigens is more frequent in the ER-negative subgroup of breast cancers, including triple-negative and basal-like breast cancers. However, expression of CT-X antigens, to our knowledge, has not been studied specifically in MBC. In studies on squamous non-small-cell lung carcinomas (33), transitional cell carcinomas of the urinary bladder (34), and gynecologic (35,36) and gastric neoplasms (37), expression of C/T antigens has been found to be correlated with patients' shorter tumor-specific survival.

It is still unclear whether C/T antigen expression contributes to tumorigenesis or represents an epiphenomenon in the process of cellular transformation related to the global genome hypomethylation (20-22,38-41) frequently occurring in highly aggressive cancers. However, our data reinforce the notion that C/T antigen specific immunization, possibly in the early stages of the disease, ie, after surgery, might be clinically relevant in selected groups of patients (19,20,23).
